# A Time-Tested Information System in Neurosurgical Oncology

**DOI:** 10.3389/fonc.2018.00593

**Published:** 2018-12-17

**Authors:** Dima Suki, David M. Wildrick, Raymond Sawaya

**Affiliations:** Department of Neurosurgery, The University of Texas MD Anderson Cancer Center, Houston, TX, United States

**Keywords:** neurosurgical oncology, database, informatics, data management, information systems

## Abstract

The Brain and Spine Center at The University of Texas MD Anderson Cancer Center is a leading multidisciplinary referral center for patients with nervous system (NS) tumors. It has a wealth of clinical experience and an internationally recognized leadership role in the management of NS cancers. In that context, an informatics infrastructure that allows the archiving of both the prospective and retrospective characterization of patients, diseases, treatments, and outcomes is invaluable. We describe our experience with the Neurosurgical Oncology Database, a database that has provided valuable, extensive, and readily searchable data on multifaceted patient, tumor, and treatment characteristics for many years, successfully serving as an administrative and operational resource and as a resource for retrospective and prospective research endeavors.

## Introduction

**Figure 1:**
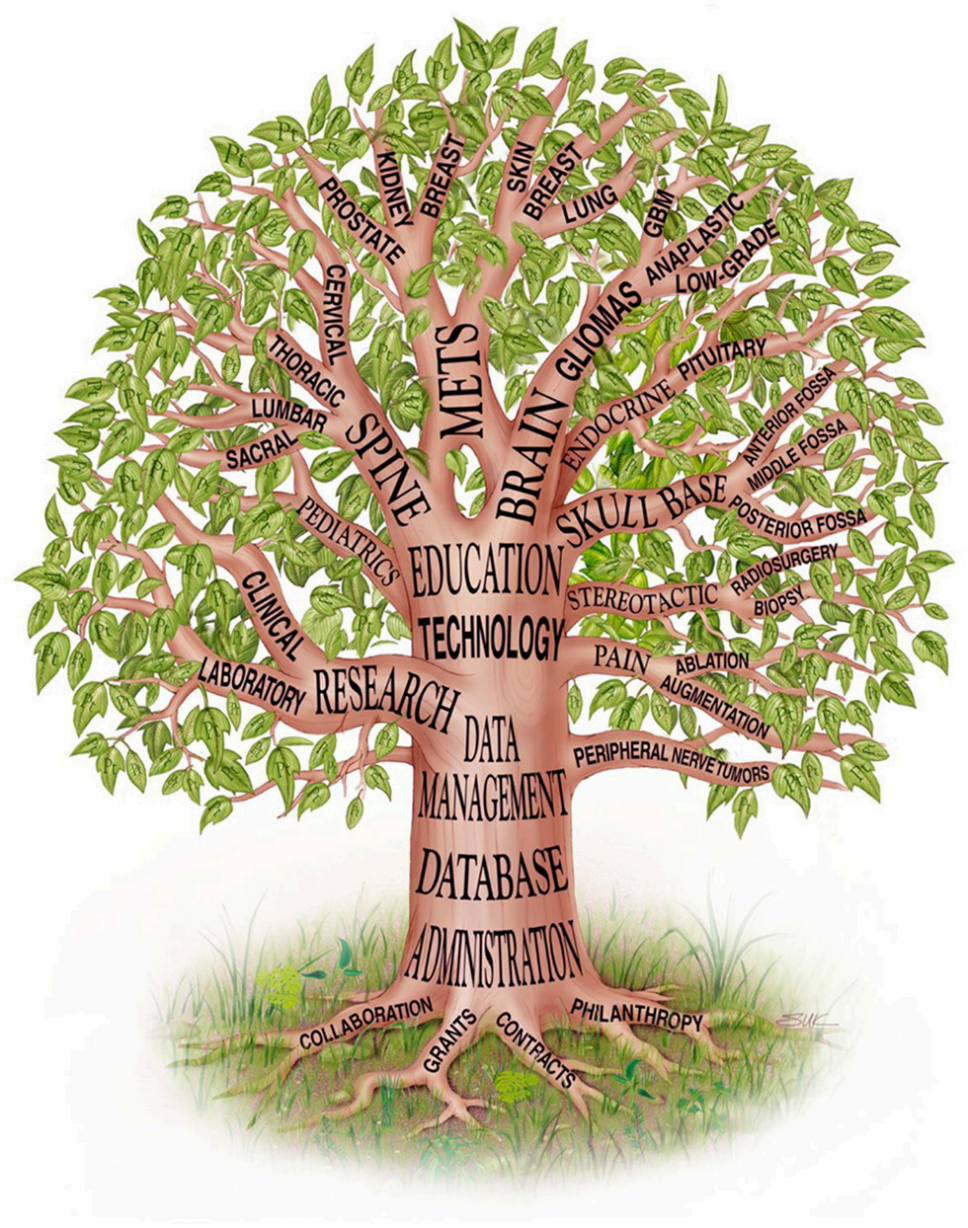
Department of Neurosurgery tree, showing the Database as a fundamental element near the base of the tree [reproduced with permission from Lang et al. (1)].

The Brain and Spine Center at The University of Texas MD Anderson Cancer Center (MD Anderson) is a major referral center for patients with nervous system (NS) tumors. It has a wealth of clinical experience and an internationally recognized leadership role in the management of NS cancers, both common and rare (1). As such, the department's leaders were keenly aware of their obligation to learn from their experience, and to apply this knowledge to improve disease classification, come up with novel therapy and management, and supply benchmark data that supports innovative experimental protocols (Figure 1). To meet this obligation, it was essential to have an informatics infrastructure that allowed us to archive and readily query both prospective and retrospective characterization of patients, diseases, treatments, and outcomes. The repository was intended to serve as an administrative and operational resource and as a resource for quality improvement, benchmarking, and educational purposes. It was also intended to serve as a valuable resource for research studies encompassing the epidemiology, natural history, characterization, and treatment of NS tumors and the multifaceted outcomes of patients with these tumors. The repository was endorsed as a top priority from day 1 and was afforded significant resources over time.

Establishing a database such as this one is a complex endeavor whose success and longevity necessitate a wide range of skills and resources on a long-term basis. This manuscript discusses the features of such a database and elements that are key to its success and longevity (Table 1).

**Table 1:** Important considerations in planning, developing and maintaining a successful database.

1. Purpose/objectives of database 2. Stakeholders/subject matter experts/users a. Intra- or interdisciplinary b. Commitment/involvement 3. Scope of database a. Target patient population b. Volume c. Disease-, site- or treatment-specific d. Cross-sectional vs. longitudinal e. Database lifetime f. Core dataset 4. Sources of data/links to other databases 5. Available infrastructure 6. Choice of database program/housing issues (interdependent with infrastructure available, issues of staffing, maintenance/enhancements/upgrades and cost) 7. Staffing a. Background/training b. Exact role c. Retention d. Cost 8. Governance plan 9. Quality assurance a. The ALCOA data integrity test (Attributable, Legible, Contemporaneous, Original and Accurate) b. Standardized data sources/definitions c. Oversight plan 10. Compliance a. Institutional review/ethical board approval b. Issues of consent/authorization vs. waiver c. Applicable policies and regulations at all levels (e.g., Code of Federal Regulations; Good Clinical Practice; Health Insurance Portability and Accountability Act [HIPAA and/or other applicable privacy laws; institutional information security policies; other policies and regulations) 11. Assessment of initial feasibility and long-term viability (baseline and maintenance costs being major considerations)

## Methods

The initial step in the development of the database was the formation of a multidisciplinary multilevel database task force. The task force was charged with identifying the uses of the database, the various data items to be collected, the format and level of detail of these items, and resolving various multidisciplinary issues. The task force included:

1 An institutional programming group. Members of this group had extensive experience in the development of complex clinical databases.
2 Committed faculty members in the various areas encompassed by the database.
3 Clinical research staff with knowledge in the NS tumor field and clinical research.
4 Data management staff that are actively involved in the data collection, verification, analysis, and reporting process.
5 A task force chairperson with extensive experience in database development and management, research protocol design and conduct, statistical analysis, regulatory compliance, as well as a strong understanding of the field of neurosciences.

A strong line of communication was secured among various team members. With the support of the task force, we applied a user-centered method to analyze tasks, workflow, and optimal interfaces for data entry, review, and mining. We then designed prototypes to map the results of user, task, and representation (interface) analyses and evaluated these prototypes. These steps were critical and fundamental to the final product.

## Hardware/Software and System Design (Figure 2)

The database is web based, which ensures easy access to users from various physical locations within the MD Anderson firewall. The software used has many of the features of other currently used data management programs, including (but not limited to) allowing complex data structures and complex logical checks. In addition, the software has superior security features and easy to navigate user and statistical software interfaces. It is backed by a premiere company and is periodically updated, thereby assuring the users state-of-the-art technology.

**Figure 2:**
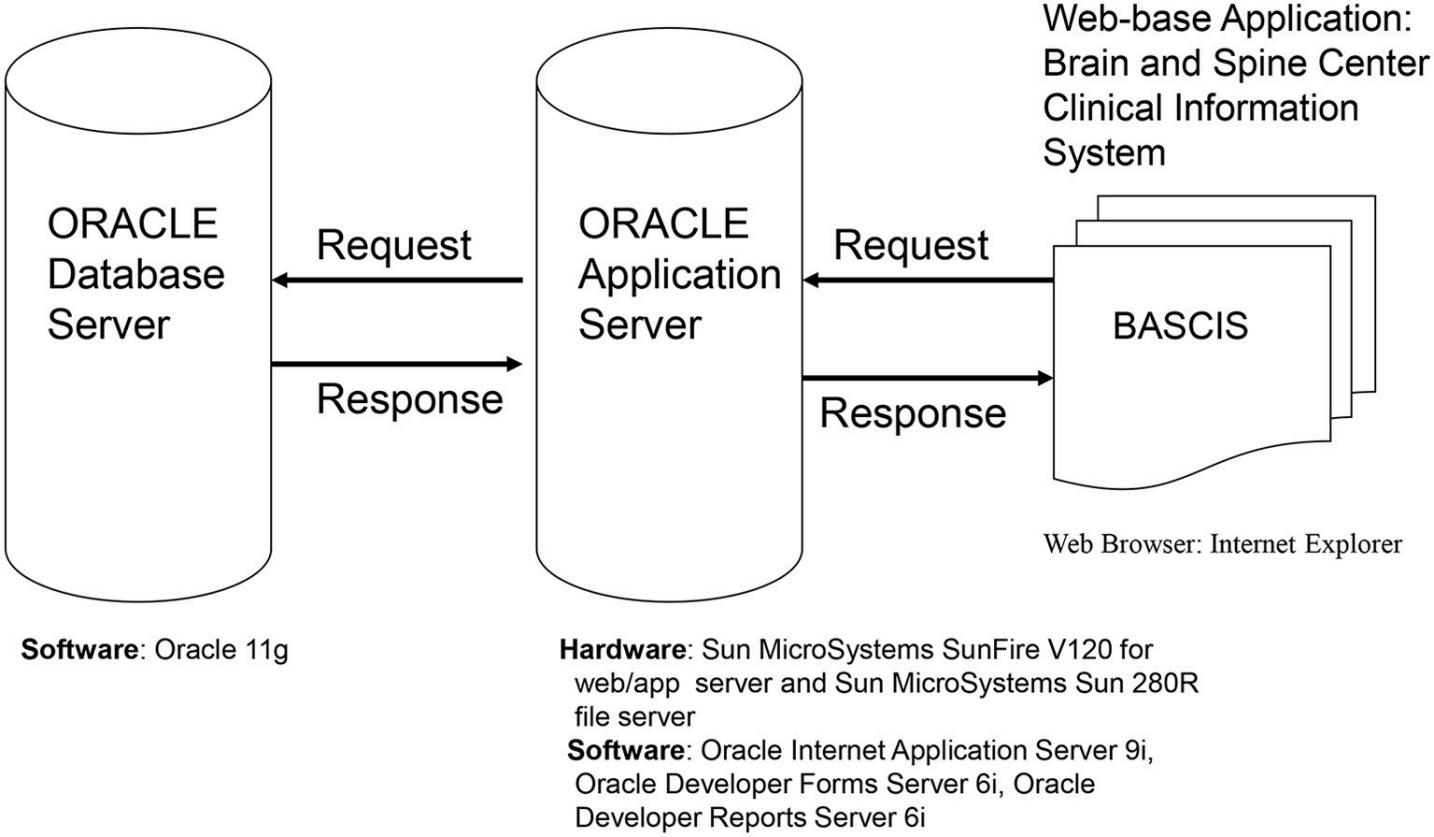
Database hardware/software and system design.

## Database Structure (Figures 3–5)

**Figure 3:**
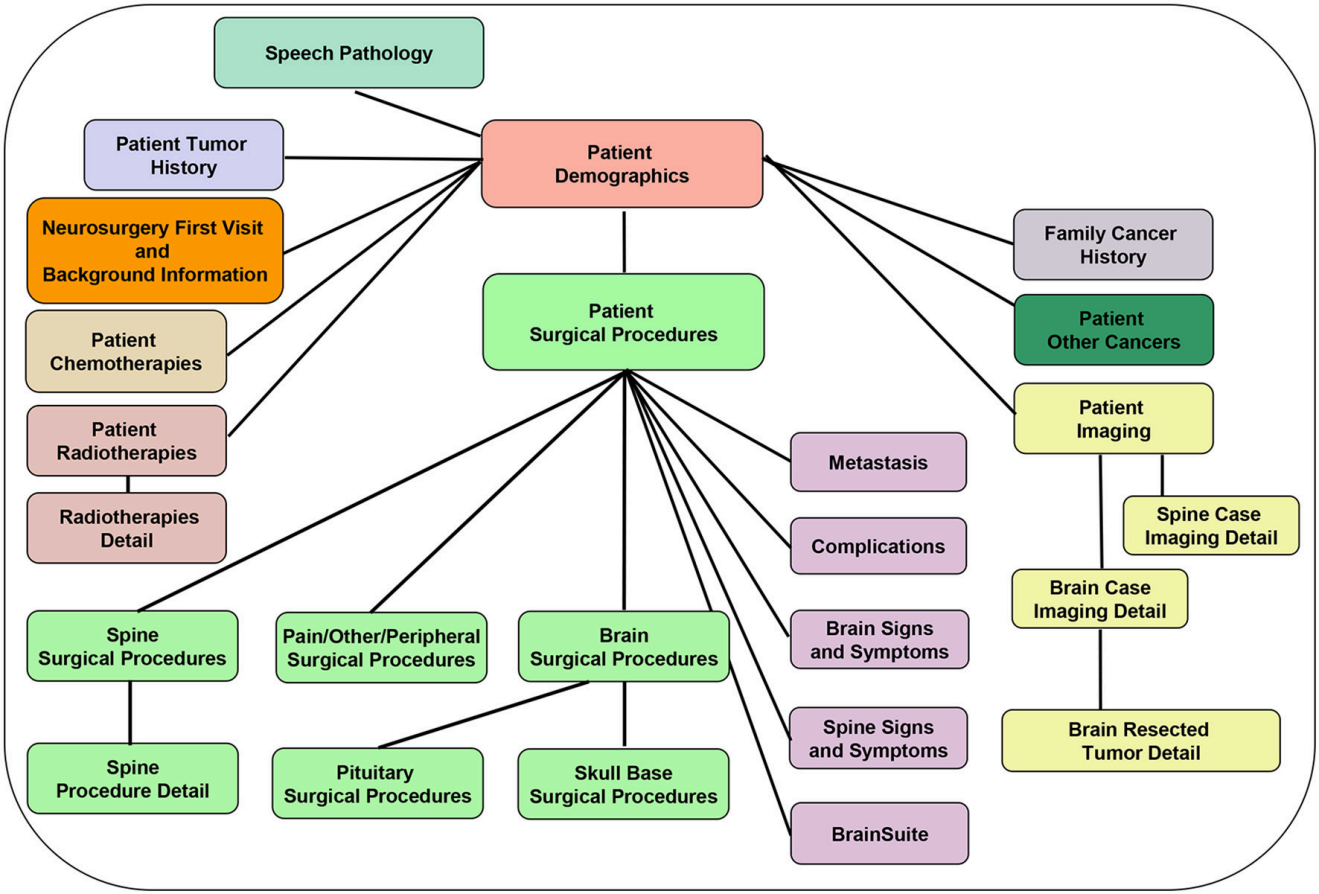
Database back-end structure, showing the various database tables and depicting the table relationships (one-to-one: e.g., Patient Demographics and Cancer Family History tables; and one-to-many: e.g., Patient demographics and Patient Surgical Procedures table).

**Figure 4:**
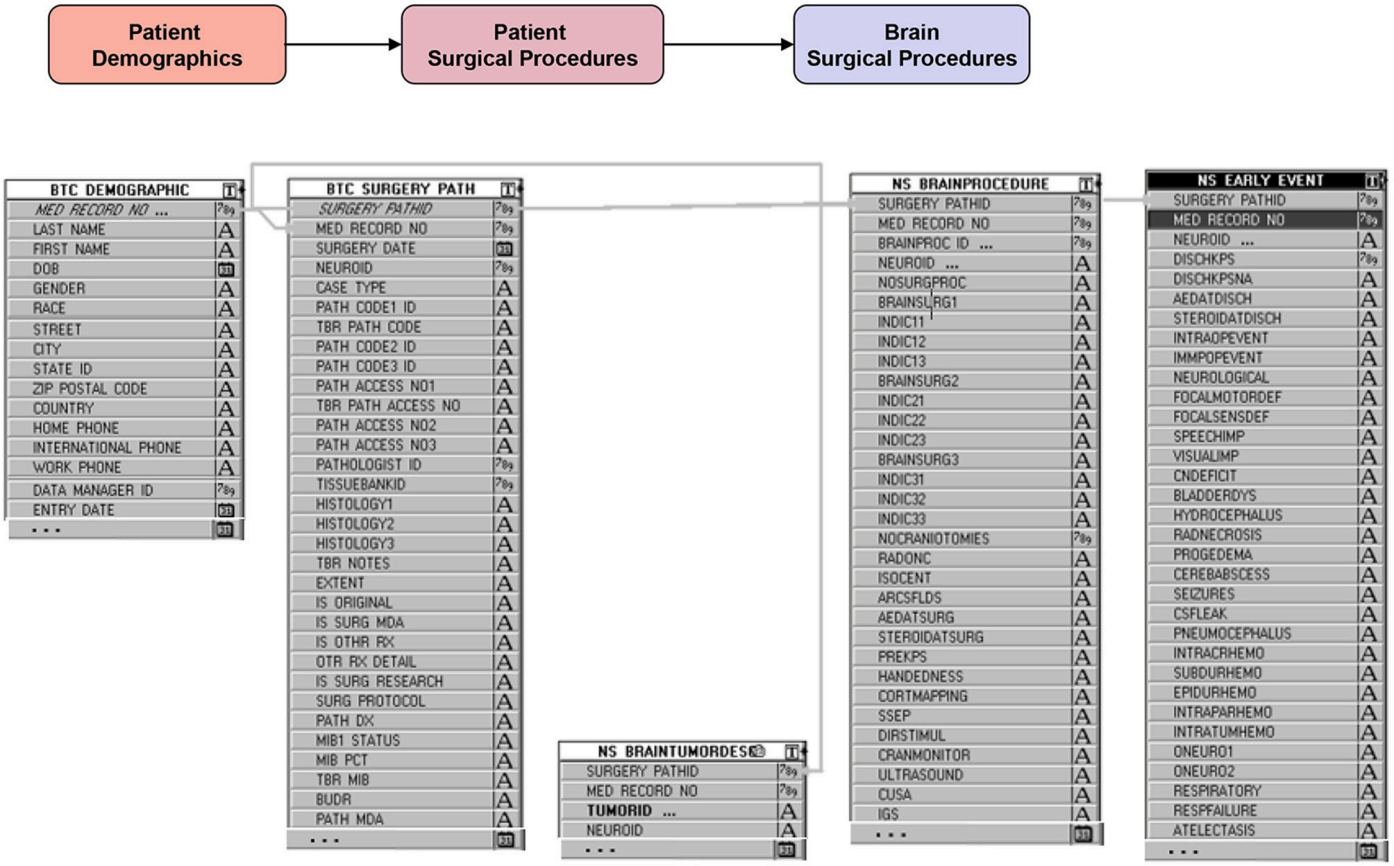
Example backend schema of brain procedure information (starting with demographic table), followed by general details of the surgery (surgery date, pathology findings, etc…), specific details of the procedures performed during the surgery and the intraoperative adjuncts used, and finally events or complications during and after surgery.

**Figure 5:**
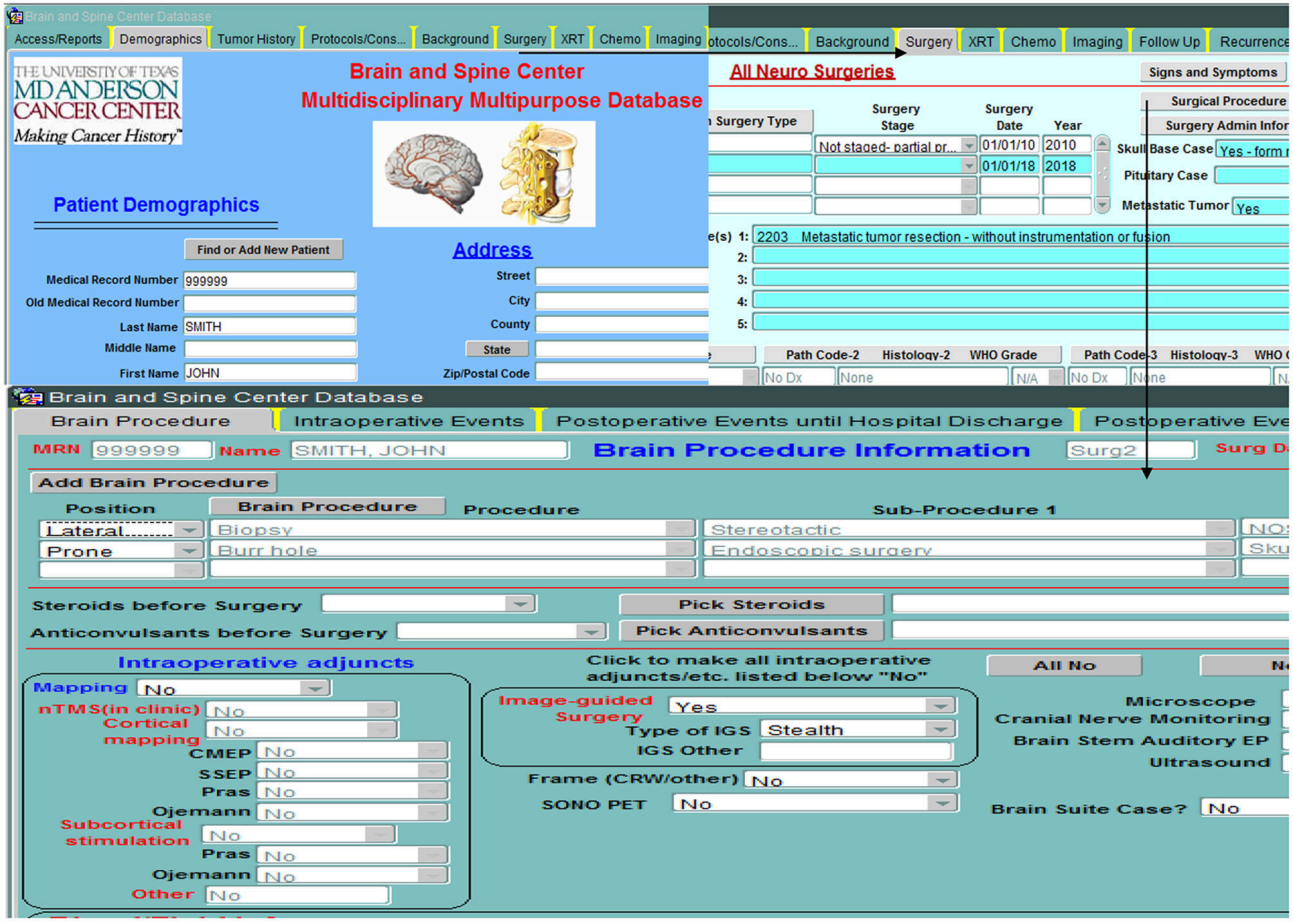
Example of brain procedure information front-end interfaces used to input data.

The database is relational, as depicted in Figure 3, and is organized into screens of varying lengths and numbers of fields. The main data entry screens are accessed through a tab at the top of the screen. Key tabs are the Demographics tab (with details on the patients' demographic information); Tumor History (with longitudinal details on the patients' tumor history, including radiographic and pathologic diagnoses, histology, grade, and metastases); Surgery (with specific sub-screens depending on the type of case (brain, skull base, spine, peripheral, pain, other; metastasis vs. primary NS; and details on all procedures related to the nervous system tumor, hospital stay, symptomatology, complications); Chemotherapy; Radiation; and Imaging. Figure 4 shows an example schema of brain procedure-related tables, and Figure 5, an example of the brain procedure interface. The database allows for additions/modifications as needed via an approved process.

## Patient Eligibility

All patients undergoing a neurosurgery at MD Anderson, irrespective of diagnosis, have their data collected and stored in the database. The requirement for patient consent was waived by the institutional review board (IRB). The waiver relied on justifications that the research (in this case strictly data collection and storage) involves no more than minimal risk to the subjects, that the waiver would not adversely affect the rights and welfare of subjects, that the collection could not be practicably carried out without the waiver, and that whenever appropriate, the subjects will be provided with additional pertinent information.

## Data Collection

The patient is registered in the database at the time of their MD Anderson neurosurgery. Registration triggers the beginning of the review/data collection process for a given patient. Dictated notes for all patient encounters (clinic visits; surgery or other procedure; testing; or other reason), scanned documents, images, and other components of the patient's electronic medical record (EMR) are reviewed after registration and until postoperative day 30 or until events and treatments during the 30 days can be captured, whichever is later. Variables entered into the database include but are not limited to: patient demographic data; clinical parameters, such as Karnofsky Performance Scale score (and other functional measures), neurologic status, symptoms, diagnosis date, cancer site(s), tumor histology (and other pathology-specific characteristics), and imaging characteristics; treatments, such as surgery details (procedures, indications, intraoperative adjuncts used, blood loss and transfusions, and extent of resection, where applicable), radiotherapy details (type, date, dose, schedule), chemotherapy details (type, date, dose, schedule); and treatment outcomes including length of hospital and rehabilitation stay, complications/toxicities, and survival time. In addition to the imaging data obtained from the patients' medical records, a qualitative visual review of the image is performed to assess for entities such as presence or absence of tumor necrosis, contrast enhancement, cysts, hemorrhage, gliomatosis cerebri, and others, as well as confirmation of tumor location and tumor functional grade. A quantitative assessment of preoperative/postoperative necrosis, tumor, and cyst volumes and extent of resection is also performed using the Vitrea software.

All patients are uniquely identified in the database. An identifier is assigned, allowing information to be retrieved without any traceable link to the actual identity of the patient (except, of course, by the database administrator/users with permission). There are multiple levels of user privilege so that various classes of users only have access to information appropriate for their roles.

Every attempt is made to collect the data in a prospective fashion, e.g., operating room findings are documented by the neurosurgeons as soon as possible after the surgery. Also, whenever possible, data acquisition from other hospital sources (e.g., hospital registration database) is automated to decrease duplication of effort, reduce error level, and ensure a quick download. Other manual downloads include data from the MD Anderson Tumor Registry, Surgical Indexing, and others. Evaluation of data from additional sources for suitability for potential download is performed on a regular basis, though not always achievable.

## Data Entry

The database contains a minimal number of text fields. Most fields are coded for consistency and ease of entry and to enhance search and retrieval capabilities. Data are entered through a point-and-click approach with drop-down menus. Entries are standardized by precise inclusion criteria and precise definitions noted in a data and database dictionary. The coding structures used have been designed for a maximum flexibility and precision of searches and data analysis. Where applicable, data on a given entity are recorded from an expandable hierarchical set of codes and linked to a number of relevant descriptors. For clarity and ease of data entry, the system was designed to show specific fields on a given screen only when applicable (e.g., fields related to the primary non-NS cancer history only appear for patients with a NS metastasis) or to automatically fill in fields in a hierarchy where appropriate. Standard diagnostic and procedural coding schemes (Systematized Nomenclature of Medicine [SNOMED and Current Procedural Terminology [CPT Systems) are also included as an additional coding methodology.

## Data Dictionary

An extensive database dictionary has been developed to serve as a reference guide for database coordinators and other database users. The dictionary includes all details on all data collected, including a description of the fields, their source, definition, and allowable responses. As a simple example: “**Any Treatment field:** Pull Down Menu options are Yes; No; Unk. This field refers to any treatment to the primary cancer at any time up to the patient's first visit to the BTC neurosurgeon. Does not include treatment to the systemic metastases. Get information from history of present illness and past medical history in neurosurgeon's dictation or previous relevant patient dictations/scan documents/or medical chart.” Additional notes or unique scenarios are highlighted as a guide for the staff. As an example: “**Note:** A family member with a primary outside the CNS that metastasized to the CNS should not be coded as one with a history of CNS cancer.”

The dictionary ensures the consistency and validity of the data stored and of their interpretation. The complete version of the dictionary is accessible within the database for easy access and is constantly updated to reflect changes.

## Database Governance

Strict written policies and procedures and standard forms are in place to govern every database-related aspect including database access and maintenance, data collection, entry, extracting, and quality assurance (Table 2). These are reviewed on a regular basis and modified as necessary. All requests for data are tracked and their status is documented.

**Table 2:** Examples of database policies and procedures in place.

◦ Account creation, deletion, modification policy and its related forms • Account creation, deletion, modification policy • Account creation form • Account modification form • Account inactivation form ◦ Change/correction process policy and form • Change/correction process policy • Change/correction request form ◦ Password change guideline ◦ Technical support procedure ◦ Neurosurgery database data capture-entry-reporting SOP ◦ Neurosurgery database QA SOP ◦ Request for data retrieval policy and its related forms • Request for individual data retrieval–research purposes • Request for data counts–research purposes • Request for data counts/retrieval–non-research purposes • Request for individual data retrieval–non-research purposes • Request for data retrieval–preparation for research purposes

## Data Queries and Retrieval (Figure 6)

The database includes some built-in reports that are routinely generated from the data. An example of such a report is the number of surgical cases during a given time frame, stratified by surgical procedure, tumor site (brain, skull base, spine, and other), surgeon, or other. The database allows the direct e-mailing of such reports to designated individuals on specified dates. Non-routine or complicated queries are performed by the departmental database programmer on an as needed basis. Reports are formatted for viewing or analysis according to the needs of the user. The database has interfaces with commonly used statistical and data management software. This allows the quick export of pertinent patient data in a standard format. Approval by designated individuals is required for all data extractions. Data to be used for research purposes will only be retrieved and distributed according to a protocol approved by the IRB.

**Figure 6:**
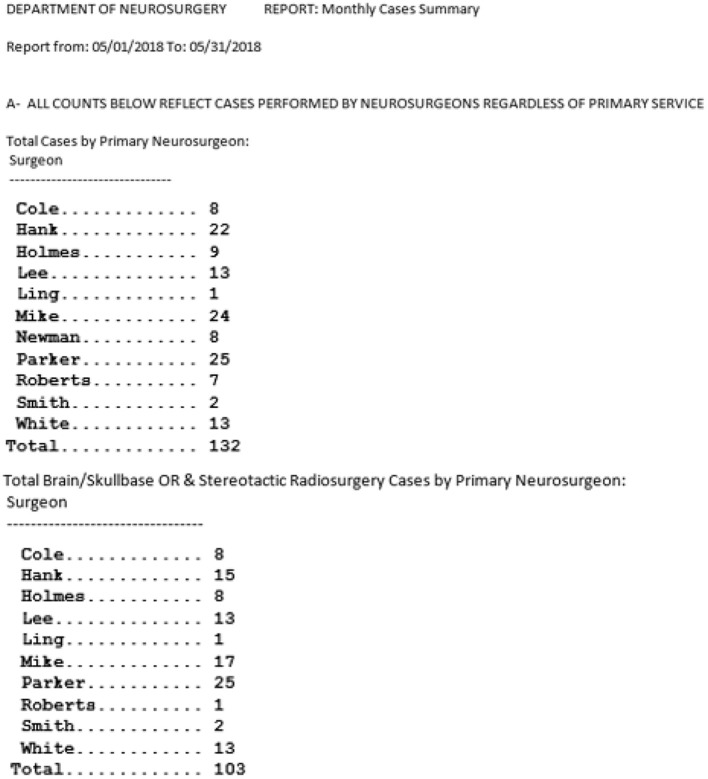
Example of basic canned (pre-programmed) data queries and retrieval interfaces: Monthly summary of cases.

## Data Quality

**Figure 7:**
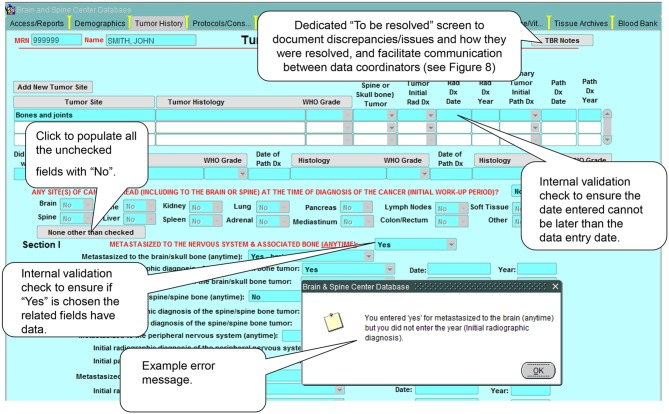
Example of built-in logic checks and validation rules. These internal consistency checks and rules help control the quality of the data in the database by preventing the input of erroneous data.

**Figure 8:**
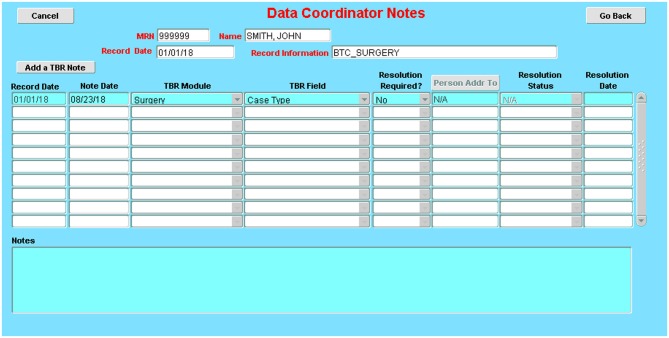
Issue-tracking sub-screen: These sub-screens, which are accessible on every database screen, allow the documentation of issues encountered during data collection and how they were resolved (e.g., error in a dictation; discrepancies between two dictations; missing details on a required field).

**Figure 9:**
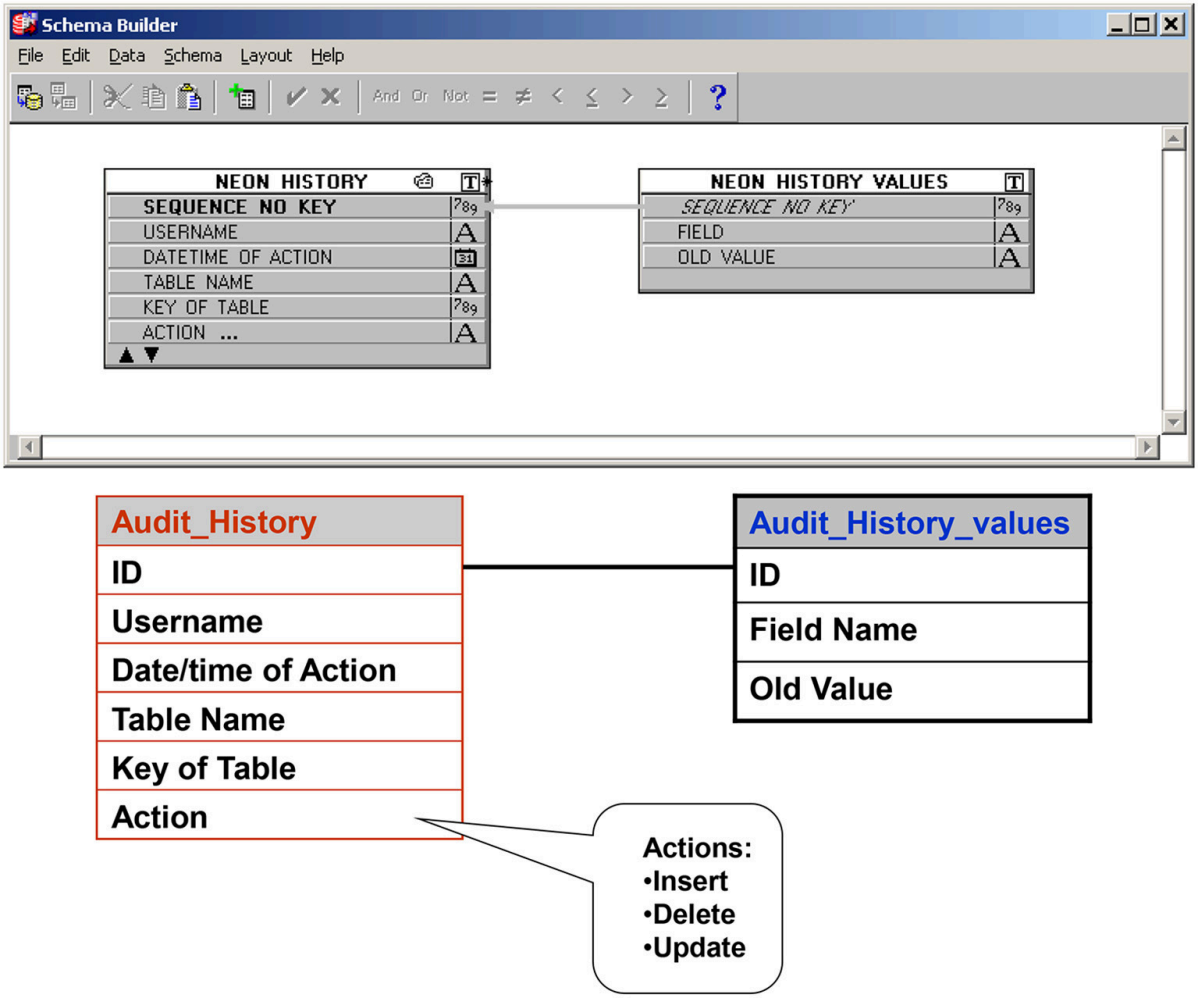
Audit trail table: Ensures that all entries (new or revisions) into the database are documented and traced to the individual who made the entry and the time the entry was made.

The most important and most difficult aspect of having a clinical database is ensuring the veracity of the data and its meeting of all applicable quality assurance (QA) standards. This issue is addressed at multiple levels:

1 Hiring of highly-qualified staff members for data extraction and coding. Given the complexity of medical data in general, and data related to the nervous system in particular, close attention was paid to the selection of the staff members, most of whom are non-practicing medical doctors, and to their training on aspects relevant to the database and data collection and coding. The database team includes 4.5 full time equivalents (FTEs) handling clinical data collection and entry, imaging, data and database management, and general oversight.
2 The data and database dictionary mentioned in the previous section, ensuring standardized and well-communicated data sources and definitions.
3 A QA standard operating procedure (SOP) detailing every aspect of the QA process as noted below. The SOP is regularly reviewed to ensure that it remains up to date and covers the following areas:
  a. A check of the daily database census against clinic and operating room schedules allows the capture of data on all eligible patients.
  b. Complex built-in logic checks and validation rules limit a large number of data entry errors/missing data at the time of entry (Figure 7).
  c. Multiple internal consistency checks of the data by designated quality assurance staff reveal missing and erroneous information not detected by the logic checks and validation rules.
  d. A dedicated “to be resolved” sub-screen on all screens allows for flagging of questionable data and signaling the need for further review by appropriate staff. (Figure 8)
  e. Comparison of a random sample of the data with the entries in the patient's medical record reveals additional errors and improves the external consistency of the data.
  f. An audit trail ensures that all entries (new or revisions) into the database are documented and traced to the individual who made the entry and the time the entry was made (Figure 9).
  g. Finally, regular staff meetings intended to go over problematic issues, determine needs for change, provide continuous training, and positively impact overall performance and outcome.

## Security and Regulatory Compliance

The database is governed by an IRB-approved protocol. Issues of patient consent and authorization are addressed as appropriate. The database meets all applicable policies and regulations at various levels (Code of Federal Regulations; Good Clinical Practice; the Health Insurance Portability and Accountability Act [HIPAA, a United States law that sets privacy standards for the protection of patients' medical records and other health information]; information security policies; other institutional policies). Particularly with regard to HIPAA compliance, the following measures are taken:

1 Access to the database is restricted to individuals with explicit permission. Users have a level of permission necessary to perform their respective jobs. Passwords need to be changed every 90 days. Application system lockout is enabled after 3 bad tries.
2 As noted above, an audit trail ensures that all entries (new/revised) into the database are documented and traced to the individual who made the entry.
3 The database contains a database-specific unique identifier that is independent of patient personal identifiers. For all requests that do not require patient identifiers, the anonymous database ID is used to identify the records. When the data are to be used for research purposes rather than for patient care or administrative reports, release of patient identifiers necessitates IRB approval. Published results obtained from any analysis are not linked to any patient identifiers.
4 The database undergoes regular check by the institutional information security department.

## Results

At the time of preparation of this manuscript, the database had spanned a period of 25 years, with the latest structure being in place for 14 years. It currently houses historical and demographic data; data on disease, tumor, and patient characteristics; and perioperative and other treatment data on close to 27,000 patients [over 34,000 neurosurgical cases with a current annual accrual of around 1,800 cases. Since its inception, the database has been highly utilized within and outside the Department of Neurosurgery for various research, administrative, educational, and other purposes. It has been the source of data for numerous publications and presentations at scientific meetings. These publications and presentations encompass areas of epidemiology, tumor characterization, treatments, and treatment outcomes. As an example, the study by Lacroix et al. (2) evaluated the outcome of 416 patients undergoing varying degrees of glioblastoma resection and required extensive use of the Neurosurgery database. It confirmed the survival advantage of a near total resection (98% or more) compared with a lesser resection. This seminal study has resulted in renewed interest in advancing neurosurgical techniques for the treatment of malignant brain tumors. In 2014, Marko et al. (3) used data from this database to study 721 patients newly diagnosed with glioblastoma (from 1993 to 2010) to construct a mathematical model of factors affecting personalized survival. Their findings argued against a surgical management strategy based on rigid extent-of-resection thresholds and instead provided the first explicit evidence supporting a maximum safe resection approach to glioblastoma surgery. These findings were further bolstered by a study by Li et al. (4), who employed our database to study the influence of maximum safe glioblastoma resection in 1,229 patients. In what is probably the largest single-center series of glioblastoma patients with extensive tumor resections, their study supported the established association between extent of resection and survival and moreover, showed that going beyond a conventional 100% resection (of all contrast-enhancing tumor) by also removing a significant portion of the fluid-attenuated inversion recovery (FLAIR) abnormality region, when safely feasible, may prolong survival without significantly increasing overall or neurological postoperative morbidity. More recently, Al-Holou et al. (5) drew data from our database on 1204 patients with glioblastoma to show that relative to piecemeal resection of these tumors, circumferential perilesional resection is significantly and independently associated with improved outcomes. Clearly, studies such as these on a scale this large would have been impossible without a patient database as robust and extensive as ours, and one that ensured that data typically inconsistently recorded in the medical records, such as method of tumor removal, or data not typically available, such as volumetric perioperative analyses of all brain tumors is regularly and consistently documented. Other select publications are listed in Table 3).

**Table 3:** Select case series publications from the neurosurgery oncology database, underscoring the opportunity to investigate the impact of novel surgical techniques and novel variables (e.g., objective measure of extent of resection, perilesional resection) on varied neurosurgical outcomes in large cohorts of patients with nervous system tumors.

**References**	**Focus**
Lacroix et al. (2)	Impact of extent of resection on the survival of patients with glioblastoma multiforme
Suki et al. (6)	Risk of leptomeningeal disease after resection or stereotactic radiosurgery for solid tumor metastasis to the posterior fossa
Suki et al. (7)	Risk of leptomeningeal dissemination of cancer after surgery or stereotactic radiosurgery for a single supratentorial solid tumor metastasis
Patel et al. (8)	Factors influencing risk of local recurrence after resection of a single brain metastasis
Marko et al. (3)	Impact of extent of resection on the survival of patients with glioblastoma mutiforme: Personalized survival modeling
Li et al. (4)	Influence of maximum safe resection of glioblastoma on survival
Al-Holou et al. (5)	Effect of perilesional resection of glioblastoma on neurosurgical outcomes
Noll et al. (9)	Relationships between tumor grade and neurocognitive functioning in patients with glioma of the left temporal lobe prior to surgical resection
Tatsui et al. (10)	Utilization of laser interstitial thermotherapy guided by real-time thermal MRI as an alternative to separation surgery in the management of spinal metastasis.
Chamoun et al. (11)	Surgical management of skull base metastases
Raza et al. (12)	Non-melanoma cutaneous cancers involving the skull base: outcomes of aggressive multimodal management.

Meetings with the data management team members are held regularly as well as on an *ad-hoc* basis. The meetings serve as a platform for dealing with problematic issues and identifying needs for modification. They aim at encouraging good data management practices and keeping communication open in a friendly unthreatening environment.

Initially, for the first few years, both an informed consent and a HIPAA authorization document needed to be signed by patients, but the IRB later approved waivers for these, given that waiver justifications for both consent and authorization were met.

Funding for the database over the years was provided by departmental funds, two MD Anderson Cancer Center institutional database grants, as well as by designated and undesignated philanthropic donor funds.

## Discussion

Information management is the creation and application of processes directed toward the collection and review of data in a structured and effective manner (13). The ready accessibility of patient, disease, treatment, and outcome data from an informatics infrastructure repository is a major catalyst for the advancement of medical knowledge, as it helps in the rapid translation of clinical and laboratory discoveries into new and better treatments and therapies. As noted under the Methods section, the database is web based, which ensures easy access to users from various physical locations protected by the MD Anderson firewall. It is sufficiently flexible to support multiple tumor and treatment types, and multiple clinical case scenarios. It incorporates elements of a prospective data collection process as well as elements of a point-of-care data entry process (including ease of access and navigation). It provides the necessary and compatible backbone for downloading data from other MD Anderson Cancer Center institutional sources. This relational database allows for user-centered, screen-driven, structured, and efficient data entry, review, and mining, and a role-based security system. It ensures the highest data quality through a large number of external and internal consistency checks, complex built-in logic checks and validation rules, dedicated “to be resolved” fields on every screen, an audit trail, a detailed data dictionary, and extensive training of qualified data management staff, a crucial prerequisite to a high quality product (14–17). It is HIPAA-compliant.

The database task force was valuable to the success of the project. Task force members brought important clinical knowledge and expertise to the group. Traditionally, clinicians may have had little or no control over the development and implementation of patient information systems. Many databases are developed by programmers with a somewhat limited understanding of the clinical issues involved. A closer look at the traditional “*Build it and they will come*” approach reveals major limitations and an inherent risk of failure. A high level of input in the development process by intended users leads to a product that meets the users' needs, has a high level of relevance, acceptance, and utilization and thereby is successful. Participation by the faculty, then, is an element crucial to the longevity of this project.

A key difficulty was the development of a robust data dictionary that clarified the various field definitions, sources, or time frames. This key difficulty was also a key strength of the database, once developed. It continues to be a living breathing document, whereby various aspects are subject to review and adaptation.

Another key difficulty was sustaining the funding. The database is an expensive endeavor, and securing financial support of full-time staff members involved in the day-to-day database activities including data collection, mining, and quality assurance is no small feat. A key strength was the hiring of highly qualified staff members and their retention. Many of the staff members have had a longevity of more than a decade with the department. During times of hardship, and these times are inevitable with a database that spans a long period of time, focus on the core data set and the “bread and butter” was crucial.

In addition to the well-known challenges of developing clinical databases in general (securing the acceptability of end-users, allowing for simple data entry and retrieval methods, and possessing large storage capacity and adequate security safeguards) (18–23), this database posed some unique challenges stemming from its unique aims. These challenges included: (1) Its wide scope (research and administrative purposes, among others); (2) the variety of tumors involved (primary vs. metastatic; brain, spine, skull base, peripheral, and each its own separate entity); and (3) the complexity and multidisciplinary nature of the management approaches, entailing differences in definitions and documentation processes and methodology.

Nevertheless, our group encompassed a wide range of expertise and valuable accumulated experiences. Both of these strengths were essential in overcoming the challenges and securing a successful endeavor. Furthermore, the perceived usefulness and relevance of the database rank high among intended users from various specialties and levels, thereby affording us strong and crucial acceptance and support necessary to overcome future challenges. Although the database represents an efficient and effective approach to handling the data management needs of a sizable multispecialty treatment center, its design and programming methodology can readily be adapted to other healthcare settings.

Single center databases such as the one described in this manuscript are invaluable in terms of the breadth and depth of information they provide and the research opportunities they present. They are crucial in providing well-annotated and consistently defined and coded data. They have a critical role to play in advancing knowledge on the topics of nervous system tumors and neurosurgical oncology and should be promoted where feasible and sustainable. That said, these databases can be hard and costly to develop and maintain (an element often underestimated during the planning phases), though the cost varies widely depending on factors such as the general infrastructure in place at the healthcare facility, the medical record system in use, the patient load, the database scope, and the caliber of the data management staff involved, to mention a few. These databases may also be limited by potential referral biases and generalization issues. National databases and registries are another valuable source of information and have allowed for valuable scientific studies. In the US, the Central Brain Tumor Registry of the United States (CBTRUS); the Surveillance, Epidemiology, and End Results (SEER); and the National Cancer Data Base (NCDB) are among the major centralized databases for brain tumor information. Each has its unique attributes, as well as its limitations: The CBTRUS includes primary tumor incidence but lacks patient follow-up data. The SEER database has incidence and follow-up data, but only on malignant brain tumors. The NCDB is the largest non-population-based US database, which identifies all newly diagnosed primary brain tumors, both benign and malignant, and has extensive patient treatment and outcome data, but because its data primarily come from hospitals accredited by the American College of Surgeons (and few unaccredited hospitals), this does not allow for tumor incidence rate estimation. None of these three public databases contain reliable or complete information on infrequently coded surgical details such as piecemeal vs. en bloc resection, dural entry and other similar variables, or outcomes such as objective postoperative tumor volume and extent of resection. In this era of big data and with the technologic advances at hand, combining data from multiple sources, single center databases, as well as national registries and databases, administrative datasets, and others, allows for a more powerful and wide-encompassing analysis of a wide array of data at every level. But an extensive discussion of this is outside the scope of this manuscript.

## Future Directions

With the advent and widespread use of the EMR, the growing wealth of electronic data encompassing all aspects of humanity, and staggering advances in technology and artificial intelligence making their way at an unprecedented and previously unimaginable pace and scope, we are pressed to pay a close look at where we are and how we should proceed in this new era. For example: (1) Wider utilization of data from various sources, such as patient-reported outcomes, the sequencing and “omics” data which have revolutionized the understanding of nervous system tumors and become essential in any research endeavor, and other data not currently in the database, and (2) linking the database to the institutional biobank will be essential to a better understanding of all relevant aspects at play. (3) Wider adaptation of available technology in an appropriate fashion to replace manual labor and connect various sources of data will decrease cost and increase efficiency. Steps that will add those features to the current ones are currently underway.

## Author Contributions

The concept of the Neurosurgical Oncology Database was originated by RS and DS. The original manuscript was written by DS, with additions to the manuscript and edits provided by both RS and DW.

### Conflict of Interest Statement

The authors declare that the research was conducted in the absence of any commercial or financial relationships that could be construed as a potential conflict of interest.
